# Brain Transcriptomic Analysis of Hereditary Cerebral Hemorrhage With Amyloidosis-Dutch Type

**DOI:** 10.3389/fnagi.2018.00102

**Published:** 2018-04-13

**Authors:** Laure Grand Moursel, Willeke M. C. van Roon-Mom, Szymon M. Kiełbasa, Hailiang Mei, Henk P. J. Buermans, Linda M. van der Graaf, Kristina M. Hettne, Emile J. de Meijer, Sjoerd G. van Duinen, Jeroen F. J. Laros, Mark A. van Buchem, Peter A. C. ‘t Hoen, Silvère M. van der Maarel, Louise van der Weerd

**Affiliations:** ^1^Department of Human Genetics, Leiden University Medical Center, Leiden, Netherlands; ^2^Department of Radiology, Leiden University Medical Center, Leiden, Netherlands; ^3^Department of Medical Statistics and Bioinformatics, Leiden University Medical Center, Leiden, Netherlands; ^4^Department of Pathology, Leiden University Medical Center, Leiden, Netherlands; ^5^Department of Clinical Genetics, Leiden University Medical Center, Leiden, Netherlands

**Keywords:** RNA sequencing and transcriptome analysis, hereditary cerebral hemorrhage with amyloidosis-Dutch type, familial cerebral amyloid angiopathy, E22Q amyloid β, E693Q mutation, extracellular matrix remodeling, mitochondrial dysfunction

## Abstract

Hereditary cerebral hemorrhage with amyloidosis-Dutch type (HCHWA-D) is an early onset hereditary form of cerebral amyloid angiopathy (CAA) caused by a point mutation resulting in an amino acid change (NP_000475.1:p.Glu693Gln) in the amyloid precursor protein (APP). Post-mortem frontal and occipital cortical brain tissue from nine patients and nine age-related controls was used for RNA sequencing to identify biological pathways affected in HCHWA-D. Although previous studies indicated that pathology is more severe in the occipital lobe in HCHWA-D compared to the frontal lobe, the current study showed similar changes in gene expression in frontal and occipital cortex and the two brain regions were pooled for further analysis. Significantly altered pathways were analyzed using gene set enrichment analysis (GSEA) on 2036 significantly differentially expressed genes. Main pathways over-represented by down-regulated genes were related to cellular aerobic respiration (including ATP synthesis and carbon metabolism) indicating a mitochondrial dysfunction. Principal up-regulated pathways were extracellular matrix (ECM)–receptor interaction and ECM proteoglycans in relation with an increase in the transforming growth factor beta (TGFβ) signaling pathway. Comparison with the publicly available dataset from pre-symptomatic APP-E693Q transgenic mice identified overlap for the ECM–receptor interaction pathway, indicating that ECM modification is an early disease specific pathomechanism.

## Introduction

Cerebral amyloid angiopathy (CAA) refers to the presence of amyloid, commonly amyloid β (Aβ), in intracerebral vessels. CAA pathology is present in the majority of Alzheimer’s disease (AD) brains and is associated with intracerebral hemorrhages in the elderly.

Hereditary cerebral hemorrhage with amyloidosis-Dutch type (HCHWA-D) is a severe monogenic form of CAA with an autosomal dominant pattern of inheritance. A point mutation at codon 693 of the amyloid precursor protein (APP) located at chromosome 21 results in a glutamine for glutamic acid substitution (NP_000475.1:p. Glu693Gln) leading to the formation of the Aβ-E22Q peptide, a toxic variant of the Aβ peptide well studied *in vitro* (Kamp et al., 2014). Pathologically, HCHWA-D is characterized by severe amyloid angiopathy of meningo-cortical blood vessels; mutation carriers suffer from intracerebral hemorrhage, starting typically between the ages of 40 and 65.

Although previous radiological and neuropathological studies describe the disease in detail, the exact molecular processes causing Aβ accumulation in the vessel wall largely remain to be characterized. Some mechanisms have been already examined in HCHWA-D post-mortem material, in particular extracellular matrix (ECM) remodeling in the angiopathic vessel wall. Changes in the basement membrane composition with accumulation of heparan sulfate proteoglycans (van Horssen et al., 2001) and activity of ECM-cross-linking enzymes like lysyl-oxidase (Wilhelmus et al., 2013) or tissue-transglutaminase (de Jager et al., 2013) are processes known to promote Aβ aggregation. More recently, our group showed a phospho-Smad 2/3-dependent dysregulation in the transforming growth factor beta (TGFβ) pathway (Grand Moursel et al., 2017) with an increase in pro-fibrotic transcripts.

Genome-wide gene expression studies are an unbiased approach to attain a comprehensive picture of dysregulated genes and pathways. RNA sequencing of post-mortem human brain tissue is of particular interest to unravel complex mechanisms in neurodegenerative diseases (Kavanagh et al., 2013), such as AD (Twine et al., 2011; Mills et al., 2013; Magistri et al., 2015). In the current study, we perform a pathway analysis on differentially expressed protein-coding genes in brain tissue of HCHWA-D patients and age-matched controls to detect potential novel therapeutic targets and biomarkers of CAA pathology and to better comprehend the molecular pathology of HCHWA-D.

Recently, the brain transcriptome of the transgenic APP-E693Q mice was published (Readhead et al., 2015), which allowed us to study overlapping pathways between our HCHWA-D patient data and pre-symptomatic APP-E693Q transgenic mice, in order to identify conserved and critical pathways in CAA.

## Patients, Materials and Methods

### Study Design

**FIGURE 1:**
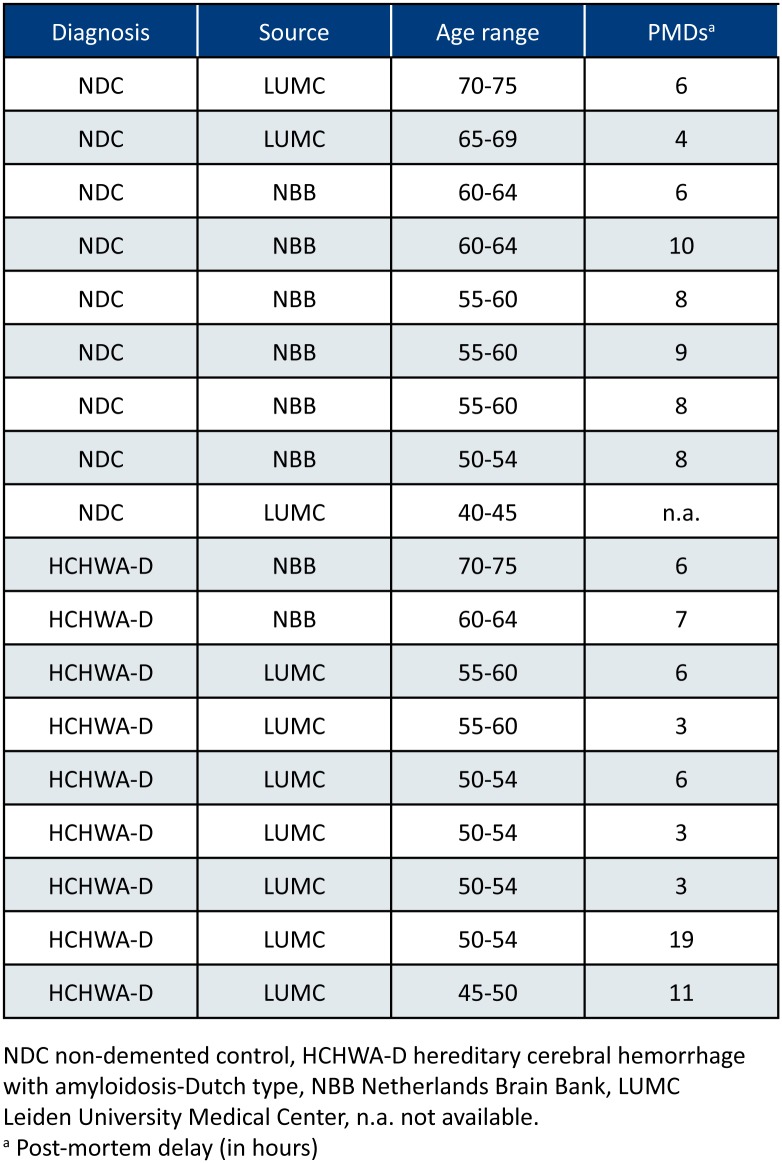
Patient material overview. Controls without stroke were age-matched [mean age ± standard deviation (SD) HCHWA-D: 55.8 ± 7.1; NDC: 58.6 ± 8.4]; both gender were included in the two groups (%M, %F; HCHWA-D: 78, 22; NDC: 56, 44); and post-mortem delays (PMDs; in hours) were not significantly different (HCHWA-D: 7.1 ± 5.2; NDC: 7.2 ± 2.2). Frontal and occipital human post-mortem brain tissue was obtained from the Netherlands Brain Bank (NBB) and from our hospital (LUMC).

This study was performed in 36 samples: frontal and occipital cortex samples from nine HCHWA-D and nine non-demented control (NDC) subjects. Patient material information can be found in **Figure 1**. Both frontal and occipital cortex were used, based on the previous finding that the CAA pathology is more severe in the occipital lobe in HCHWA-D compared to the frontal lobe (Maat-schieman et al., 2005). Written informed consent was obtained for each donor in accordance with the Declaration of Helsinki and all material and data were handled in a coded fashion maintaining patient anonymity according to Dutch national ethical guidelines (Code for Proper Secondary Use of Human Tissue, Dutch Federation of Medical Scientific Societies). The study was approved by the local Ethics Committee (Commissie Medische Ethiek, LUMC).

### RNA Isolation, Library Preparation, and Sequencing

Brain tissue processing, tissue homogenization, and RNA extraction were done as described previously (Grand Moursel et al., 2017; see Datasheet 1). Samples for RNA extraction were processed directly, whereas samples for protein extraction were stored at -80°C prior to analysis. RNA content was measured with the Nanodrop at 260 nm and evaluation of RNA integrity was performed with on-chip electrophoresis using an RNA 6000 Nano kit and a Bio-Analyzer 2100 (Agilent). Samples with an RNA integrity number (RIN) value of <5 were excluded from the study.

RNA samples were depleted for ribosomal RNA (rRNA) with the Ribo Zero Gold Human kit (Illumina) and strand specific RNA-Seq libraries were generated as previously described (Parkhomchuk et al., 2009), with minor modifications as defined in Datasheet 1. After amplification of the libraries, samples with unique sample indexes were pooled and sequenced paired-end 2x50bp on a HiSeq 2500 system following standard Illumina guidelines.

### Mapping Reads, Gene Expression Counts, and Quality Controls

RNA-Seq files were processed using the BIOPET Gentrap pipeline v0.6 developed at the LUMC^1^ which performs FASTQ preprocessing (including quality control, quality trimming, and adapter clipping), RNA-Seq alignment, read and base quantification, and optionally transcript assembly. FastQC (RRID:SCR_000141, v0.11.2) was used for checking raw read QC. Low-quality read trimming was done using Sickle (RRID:SCR_006800, v1.33) with default settings. Adapter clipping was performed using Cutadapt (RRID:SCR_011841, v1.9.1) with default settings. RNA-Seq reads’ alignment was performed using GSNAP (RRID:SCR_005483, v2014-12-23) with setting “–npaths 1” on GRCh38 reference genome without the alternative contigs. The gene read quantification was performed using HTSeq-count (RRID:SCR_011867, v0.6.1p1) with setting “–stranded reverse.” The gene annotation used for quantification was UCSC RefSeq genes for GRCh38 downloaded on 2015-07-13.

SNPs and gender-based quality controls as well as median 5′–3′ bias methods are specified in Datasheet 1.

### Normalization and Differential Expression Analysis

CQN (RRID:SCR_001786, v1.22) was used to normalize the gene count table for library sizes, gene transcript lengths (sum of exons lengths), and gene transcript GC-contents (concatenated exon sequences). EdgeR (RRID:SCR_012802, v3.18.1) was used to perform differential gene expression analysis. An interaction model *group^∗^area* was fit with two factors: *group* (two categories: HCHWA-D vs. control) and *area* (two categories: frontal vs. occipital). As offsets we used the normalization coefficients obtained from the CQN model. Benjamini and Hochberg FDR was computed to adjust *p*-values obtained for each differentially expressed gene (DEG). Finally, a table of raw and normalized expression levels of the genes was produced (not shown), and a table of gene expression levels fold changes (FCs) and their significances for each gene and model component.

### Validation of RNA-Seq Data

Quantitative RT-PCR (qPCR) was performed with the same RNA extracts as were used for RNA-Seq analysis and the primers listed in Datasheet 2. Primer design, qPCR method, and analysis that were done as described previously (Grand Moursel et al., 2017) are provided in Datasheet 1. Correlation of expression levels between the RNA-Seq and the qPCR data was calculated from expression levels of selected genes extracted from the count matrix. The gene expression mean values per patient (log2 transformed) of both the count matrix and the qPCR results were plotted.

Western blot was performed from frozen brain sections as described in Datasheet 1 with anti-HSP70 (1:1500, Santa Cruz Biotechnology Cat# sc-24, RRID:AB_627760) and anti-β-actin (1:5000, Abcam Cat# ab6276, RRID:AB_2223210).

All statistical analyses from the validation section were conducted in GraphPad Prism (RRID:SCR_002798, v7.00) with a level of statistical significance set at *p* < 0.05.

### Pathway Analysis

Gene ontology (GO) annotations and significantly altered pathways in KEGG, Reactome, and Wikipathways were analyzed using GeneTrail2 (Stöckel et al., 2016; RRID:SCR_006250, v1.5; GO, KEGG, Reactome retrieved 11/01/2016; Wikipathways retrieved 18/02/2016) with the gene set enrichment analysis (GSEA) method. GSEA was performed using a Kolmogorov–Smirnov non-parametric rank statistic (Backes et al., 2007) with Benjamini and Yekutieli FDR multiple testing adjustment method. Gene lists were ranked based on either FC or FDR (top list with most significantly up-regulated and bottom list with most significantly down-regulated; keeping the dysregulation direction).

**FIGURE 2:**
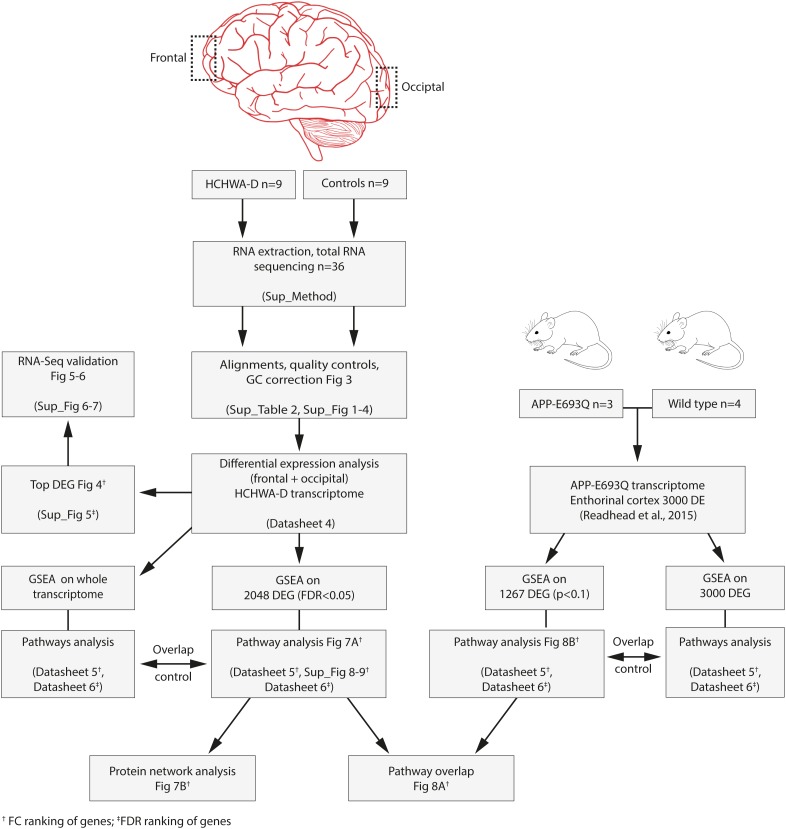
Flowchart of the study and associated files.

Analysis was performed on extended gene sets and most significant DEGs from HCHWA-D brains from the current study and from transgenic APP-E693Q mice (DEG WT vs. APP-E693Q mice, entorhinal cortex (Readhead et al., 2015) as depicted in **Figure 2**. Subsequent known interactions between genes set were visualized with the STRING database (RRID:SCR_005223, v10.5).

## Results

### Quality Checks of Samples and RNA-Seq Reads

Based on SNPs, gender, and HCHWA-D mutation presence, all 36 samples were concordant and included in the analysis (Datasheet 3). The average number of reads after sequencing was 19,578,485 for controls and 23,038,641 for HCHWA-D. The number of reads after alignment is depicted in Datasheet 3. The count matrix was generated with uniquely mapped reads. On average, the HCHWA-D samples had a lower RIN value than control samples (6.50 ± 0.96 and 7.92 ± 0.78, respectively). The median 5′–3′ bias (ratio of median 5′bias:3′bias) was calculated for each sample (samples′ code and details in Datasheet 2). High values (>5) were found for three control samples (S_7, S_17, and S_18) and eight HCHWA-D samples (S_19, S_21, S_22, S_23, S_24, S_28, S_32, and S_36), but we did not find a significant correlation with a longer post-mortem delay (PMD) or a lower RIN value (not shown). On the other hand, there was a strong positive correlation between the median 5′–3′ bias and the number of reads with GC-content > 75% (Datasheet 3). This prompted us to apply a GC correction before analyzing DEGs.

### Quality Controls of Differential Gene Expression and Analysis

**FIGURE 3:**
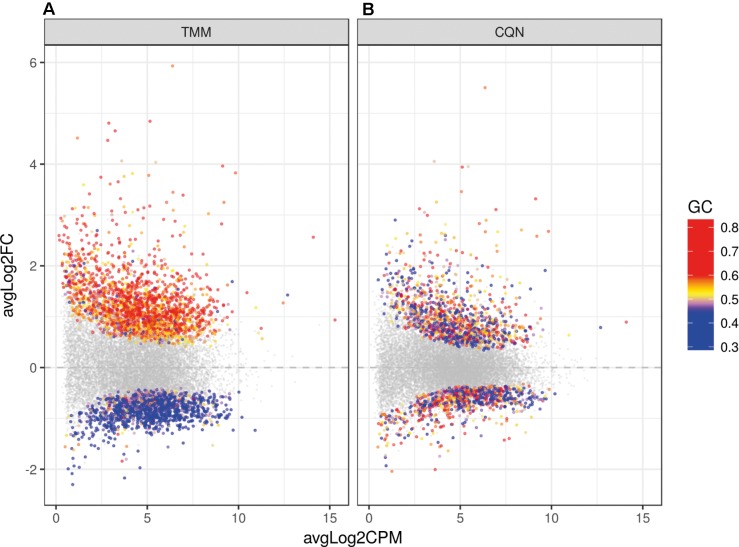
Dot plot of the differentially expressed genes (DEGs) in HCHWA-D samples compared to the control samples with log2 fold change (log2FC) vs. log2 counts per million (log2CPM) **(A)** without correction and **(B)** with CQN correction for GC bias. Significant DEGs in both panels are highlighted by colored dots; colors in legend indicate the GC-content.

Because of large differences in the GC-content of samples, correction for GC bias was done with the CQN model. After GC correction, FCs′ dependence of gene GC-contents was no longer observed (**Figure 3**). The clustering of reads was examined with principal component analysis (PCA) and no clustering related to the gender or the origin of tissue (NBB vs. LUMC; data not shown) was identified. PCA showed a close clustering of the control samples with less homogeneity in the HCHWA-D samples. Most frontal and occipital samples from the same subject clustered together (Datasheet 3).

**FIGURE 4:**
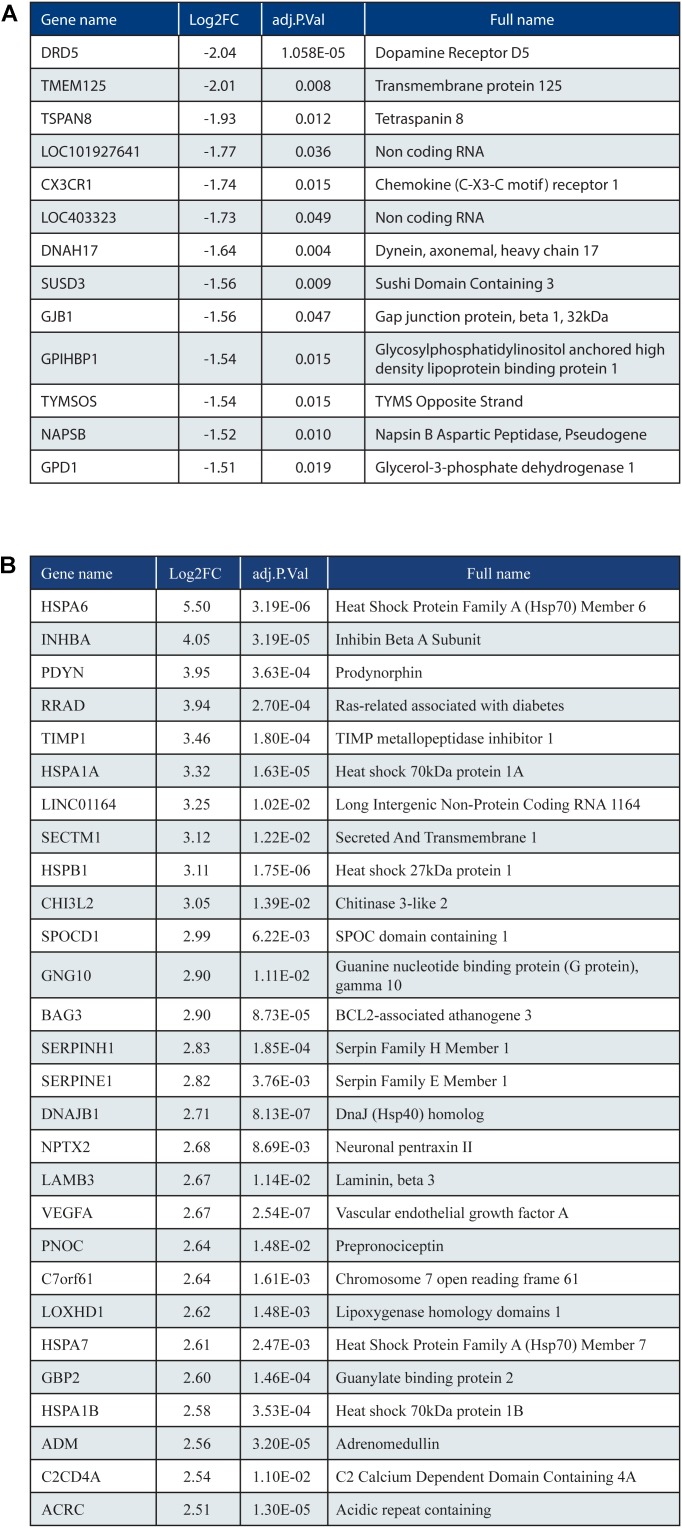
**(A)** Top list of down-regulated genes based on fold change (ranking based on log2FC < –1.5; 13 out of 847 genes shown). **(B)** Top list of up-regulated genes (ranking based on log2FC > 2.5; 28 out of 1201 genes shown).

When we analyzed DEGs independently of cases, we found 380 genes differently expressed in frontal vs. occipital cortex, but in the HCHWA-D cortex, we did not find evidence that the two brain regions were differently affected. Therefore, in all further analyses, frontal and cortical samples in each group were pooled (18 vs. 18 samples). HCHWA-D whole dataset and subset of significantly altered genes (FDR < 0.05) with both FC and FDR are provided in Datasheet 4. A total of 2048 significant DEGs were identified (**Figure 3B**) including 1201 (7.5%) up-regulated DEGs and 847 (5.3%) down-regulated DEGs. The top list of DEG, ranked on FC, is given in **Figure 4A** (down-regulated DEG with both FC and FDR) and **Figure 4B** (up-regulated DEG with both FC and FDR). Top DEG ranked on significance can be found in Datasheet 3.

### Validation of Top Up-Regulated and Down-Regulated Genes

**FIGURE 5:**
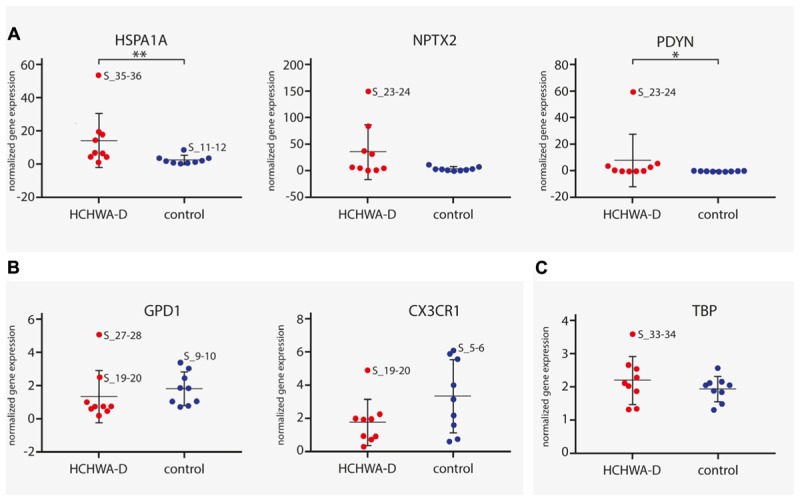
qPCR analysis of **(A)** three up-regulated genes (*HSPA1A*, *NPTX2*, and *PDYN*) and **(B)** two down-regulated genes (*GPD1* and *CX3CR1*). Transcript levels of HCHWA-D samples were not following a normal distribution (data not shown). *HSPA1A* and *PDYN* were found significantly up-regulated (MW test, *p* = 0.006^∗∗^ and *p* = 0.040^∗^, respectively). For *GPD1*, significance was reached upon removal of the greatest outlier (*t*-test, *p* = 0.030^∗^; S_27-28 outlier identified with the ROUT method, *Q* = 1%; data not shown), no significant outliers found for *CX3CR1*. Transcript expression levels were normalized with two reference gene, *n* = 9. **(C)** *TBP* normalization control was not significantly different (*t*-test, *p* = 0.32). ^∗^*p* < 0.05 and ^∗∗^*p* < 0.01. RNA-seq samples code are used to identify greatest outlier.

Five genes from the top DEG (up- and down-regulated) were selected based on FC, FDR, and expression level. Three up-regulated genes (*HSPA1A*, *NPTX2*, and *PDYN*) and two down-regulated genes (*GPD1* and *CX3CR1*) were validated by qPCR. The trend for up- or down-regulation was confirmed, although statistical significance was not reached for every target principally due to high variability in patient samples (**Figure 5**). Nevertheless, the correlation of gene expression per patient between the count matrix (log2CPM) and the qPCR was highly significant for *NPTX2*, *PDYN*, and *GPD1* and significant for *HSPA1* and *CX3CR1* (Datasheet 3).

**FIGURE 6:**
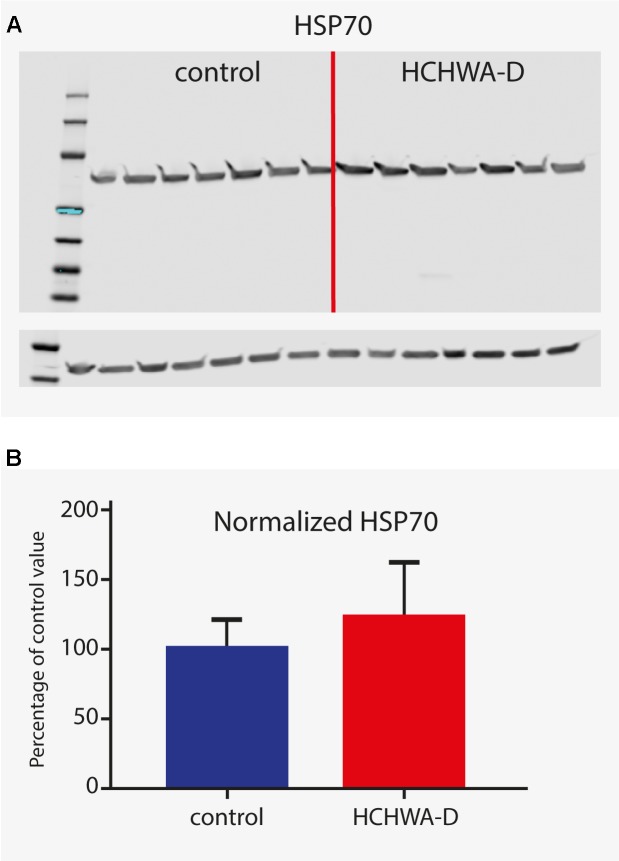
Western blot analysis of HSP70 (antibody detecting *HSPA1A* and *HSPA1B*). **(A)** Signals given by HSP70 antibody showing a band at 70 kDa (with β-actin loading control under main blot). **(B)** Signal intensity quantification (*n* = 7, β-actin normalized). Higher protein level in HCHWA-D occipital cortex compared to control occipital cortex was measured although not significantly different; *p* = 0.1888 with a two-tailed unpaired Student’s *t*-test.

For protein level validation of these five genes, western blots were performed on brain homogenates. HSP70 protein was found to be up-regulated in occipital cortex of HCHWA-D, although the difference was not significant (**Figure 6**; original picture in Datasheet 3).

### Pathway Analysis

We performed GSEA in Gene Trail2 on gene sets sorted by FC. GSEA tests per category whether the genes in the set are randomly distributed or accumulate at the top (“enriched” pathway) or the bottom (“depleted” pathway) of the sorted input list (Backes et al., 2007). Accordingly, “depleted” pathways are over-represented by down-regulated genes and “enriched” pathways are over-represented by up-regulated genes. Although GSEA does not require pre-processed expression data, it has been used on thresholded data, for example, to identify robust molecular signatures in tumor diagnosis (Monti et al., 2005). Similarly, in order to identify predominant pathways and genes dysregulation caused by the APP p.Glu693Gln substitution in HCHWA-D, we conducted analysis both on the whole dataset and on the subset of significantly altered genes. Top annotated GO processes were similar in both analyses for the HCHWA-D dataset and for the murine dataset (exhaustive analysis in Datasheet 5 for genes ranked on FC). The GSEA on genes ranked on FDR is provided in Datasheet 6. The analysis of the subset of significantly altered genes (ranked on FC) is detailed below.

#### GSEA on HCHWA-D Dataset

**FIGURE 7:**
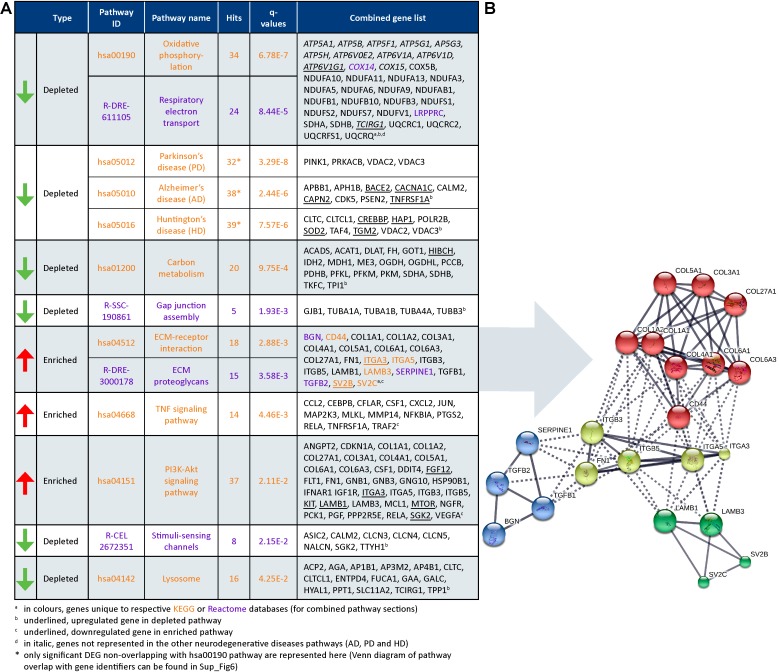
Dysregulated pathways in HCHWA-D. **(A)** Significantly dysregulated pathways in HCHWA-D DEG subset and associated genes identifiers in GeneTrail2 v1.5 (with fold change input; ranked on statistical significance except for the combined HD, AD, and PD categories). Depleted pathways (green arrows) are over-represented by down-regulated genes and enriched pathways (red arrows) are over-represented by up-regulated genes. Text color codes distinguish pathways originating from KEGG (orange) or Reactome (purple) databases. **(B)** Schematic representation of known interactions between genes of the ECM-related pathways. Representation of high confidence interactions with MCL clustering in String interaction database (line thickness indicates the strength of data support).

Gene set enrichment analysis on the significant DEG subset (FDR < 0.05; 2036 out of 2048 genes were recognized) was performed. Top annotated GO processes were associated to “depleted” mitochondria-related categories and “enriched” ECM-related categories. Major dysregulated pathways and identified genes in the DEG subset from Reactome and KEGG are summarized in **Figure 7A**.

Cellular respiration pathways (oxidative phosphorylation and respiratory electron transport) were “depleted” as well as the neurodegenerative diseases pathway in KEGG [AD, Parkinson disease (PD), and Huntington disease (HD)]. As most genes from these pathways overlapped with the cellular respiration (Datasheet 3), only the genes non-related to cellular respiration were included in the PD, AD, and HD categories in **Figure 7A**. Oxidative phosphorylation genes specific for HCHWA-D, i.e., not represented in the other neurodegenerative disease categories were the ATP6V subunits, *COX14*, *COX15*, *LRPPRC*, and *TCIRG1*.

Extracellular matrix-related pathways (ECM–receptor interaction and ECM proteoglycans) were significantly “enriched” pathways. Expression values boxplots (Datasheet 3) show that many of these genes follow highly concordant trends. Four clusters were defined based on known interactions between genes in this group (**Figure 7B**). The main cluster (red) includes *CD44* and multiple collagen genes. *CD44* has direct interactions with the three other clusters: integrins and fibronectin (*FN1*) cluster (yellow), laminin B and SV2 cluster (green), and TGFβ (*TGFB1* and *TGFB2*), serpin family E member 1 [*SERPINE1* also known as plasminogen activator inhibitor-1 (PAI-1)], and biglycan (*BGN*) cluster (blue). The integrin and fibronectin groups have a central role with interactions with the other three clusters. Additional correlation plots of the gene expression levels per patient between RNA-Seq and qPCR data are given for *TGFB1*, *TGFBR2*, *FN1*, *SERPINE1*, *TIMP-1*, and *Col1A1* pro-fibrotic genes (Datasheet 3).

#### GSEA on APP-E693Q Dataset and Pathways Comparison

**FIGURE 8:**
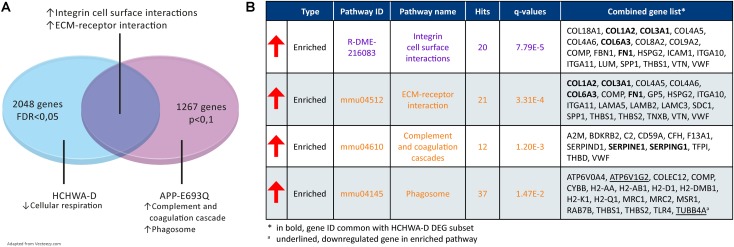
Transcriptome comparison of HCHWA-D and APP-E963Q mouse model (entorhinal cortex; APP-E693Q vs. WT; Readhead et al., 2015). **(A)** Venn diagram depicting major dysregulations and overlap in enriched pathway. **(B)** Significantly dysregulated pathways in APP-E693Q DEG subset and associated genes identifiers in GeneTrail2 v1.5 (with fold change input; ranked on statistical significance). Text color codes distinguish pathways originating from KEGG (orange) or Reactome (purple) databases.

Murine DEGs (entorhinal cortex; APP-E693Q vs. WT; Readhead et al., 2015) were extracted and GSEA on a DEG subset (*p* < 0.1; 1088 out of 1267 genes were recognized) was performed. A transcriptomic comparison between HCHWA-D patients and APP-E693Q mice at the pathway level is schematically shown in **Figure 8A**. Major dysregulated pathways and identified genes in the APP-E693Q subset are summarized in **Figure 8B**.

## Discussion

Our study provides a comprehensive transcriptome analysis of human HCHWA-D brain cortex. Using RNA-Seq, we identified oxidative phosphorylation dysfunction and enrichment in ECM-related pathways as major transcriptomic changes in HCHWA-D and we revealed an overlap in affected pathways with the APP-E693Q mouse model.

### Homogeneity in Gene Expression Between Cortical Regions

In the current report, we did not find evidence that frontal and occipital cortexes of HCHWA-D were differently affected. This finding is in agreement with our previous study where we found no difference in CAA severity between occipital and frontal lobes, both area being similarly severely affected at pathological examination (Grand Moursel et al., 2017).

### RNA Quality in HCHWA-D Group and Corrections

Although the RIN values on average were lower in the HCHWA-D group, we did not observe an effect of the RIN value on the number of reads, and therefore no correction for RIN value was applied. This is supported by a study of human post-mortem brain *ex vivo* degradation (mimicking PMD) where correcting for RIN value in data analysis did not remove the induced degradation bias (Jaffe et al., 2016). Nevertheless, a GC bias positively correlated with the median 5′–3′ bias, and affecting the FC after normalization, was corrected. Additionally, GC-content of genes might be associated with RNA decay and genes playing regulatory function were found more unstable (Gallego Romero et al., 2014; Feng et al., 2015; Jaffe et al., 2016). Alternatively, lower RNA quality was recently associated with dementia diagnostic (Miller et al., 2017), also occurring in HCHWA-D (Wattendorff et al., 1995; unknown dementia status here). Accordingly, an accelerated RNA degradation in HCHWA-D samples could reflect underlying pathogenesis and brain damage. We are confident that the GC correction rectified possible RNA integrity differences as we found a very high correlation with qPCR on random-primed cDNA, a technique that suffers much less from RNA degradation and GC bias.

### Oxidative Phosphorylation Dysfunction in HCHWA-D

Cellular aerobic respiration, including ATP synthesis and carbon metabolism (TCA cycle and glycolysis/gluconeogenesis), were most significantly altered pathways, dominated by down-regulated genes, which indicates a mitochondrial dysfunction in HCHWA-D.

The mitochondrial respiratory chain complex I (NADH-ubiquinone) and the ATP synthase complex V (F-type ATPase) were altered pathways in HCHWA-D like in other neurodegenerative diseases (AD, PD, and HD). This cellular energy dysfunction is a common denominator in neurodegenerative diseases (Lin and Beal, 2006; Golpich et al., 2017). Mostly studied in AD, in the context of neuronal mitochondrial dysfunction (Costa et al., 2012), oxidative injury has also been studied in endothelial and perivascular cells (Di Marco et al., 2015). In particular, *in vitro* studies have shown that especially the Dutch-Aβ-peptide (AβE22Q), via accelerated generation of toxic oligomeric species, could induce mitochondrial alteration of smooth muscle and endothelial cells, leading to induction of apoptosis (Ghiso et al., 2014). Although confirmed in the APP-E693Q mice, the presence of oligomeric Aβ species in HCHWA-D has not been demonstrated yet.

The vacuolar proton pumps (V-type ATPase), responsible for acidifying the vacuolar system and in particular in the phagosomal/lysosomal vesicles, was also part of the oxidative phosphorylation pathway affected in HCHWA-D (ATP6V subunits and *TCIRG1*). Since vacuolar proton pumps have a high ATP demand, mitochondrial dysfunction thus could also impact autophagocytosis and protein turnover, thereby contributing to pathogenic protein accumulation (Ganguly et al., 2017).

### Enrichment in ECM-Related Pathways in HCHWA-D

In contrast, genes in the ECM-related pathways were mostly up-regulated in HCHWA-D brain samples. We showed differential expression of *CD44*, a cell-surface glycoprotein that acts as a principal receptor for ECM proteins such as hyaluronic acid, osteopontin, and collagens and mediates the cell surface activation of matrix metalloproteinase. *CD44* has been implicated in several inflammatory diseases (Misra et al., 2015), as well as in AD pathology where an increase in reactive CD44-positive astrocytes was demonstrated (Akiyama et al., 1993). In addition, CD44v6 and CD44v10 splice variants were significantly higher in AD hippocampal neurons (Pinner et al., 2017), and in lung fibrosis, CD44v6 is mediating the induction of *COL1* pro-fibrotic action of TGFβ1 (Ghatak et al., 2014). In HCHWA-D, up-regulation of *FN1*, *SERPINE1*, and collagen genes also suggests TGFβ1-mediated fibrosis, as proposed in our recent study (Grand Moursel et al., 2017). The presence of *TIMP-1* among the top five up-regulated genes strengthens this hypothesis. *TIMP-1* is a strong inhibitor of matrix metalloproteinase, thereby reducing ECM turnover and exacerbating the fibrosis. Of interest, *FN1*, which was commonly up-regulated in HCHWA-D and APP-E693Q mice, was also found up-regulated in plasma of both asymptomatic HCHWA-D and *PSEN1* mutation carriers (Muenchhoff et al., 2016) and was recently proposed as a blood-based biomarker for AD (Long et al., 2016). Additionally, basement membrane thickening of the vessel wall by *FN1* was shown in early stages of AD before Aβ deposition (Lepelletier et al., 2015). The use of ECM component detection in plasma, like *FN1*, as biomarkers to monitor early CAA-associated changes in the cerebrovasculature requires further investigation. Of therapeutic relevance, *PSTG2* (COX-2) inhibitor celecoxib was found to lower *FN1* and *COL1* expression and attenuate the vessel wall thickness in stroke-prone spontaneously hypertensive rats (Tang et al., 2015).

### Other Identified Pathways in HCHWA-D

Amyloid precursor protein expression was not up-regulated, but some specific APP cleavage enzyme genes were altered, indicating disturbances in APP processing. *APHB1* and *PSEN2*, both functional component of the gamma-secretase complex (cleavage at Aβ C-terminal side) were down-regulated, possibly also affecting the Notch-signaling. *BACE2* (cleavage at β-site and within Aβ region), on the other hand, was up-regulated. Aβ production by *BACE2* was increased by the Flemish mutation (p.AlaA692Gly) and *BACE2* is highly expressed in the vasculature (Farzan et al., 2000). These findings are in accordance with earlier studies suggesting that APP-Dutch mutation pathogenic effect was due to an altered APP processing more than a global increase in Aβ production (De Jonghe et al., 1998; Watson et al., 1999; Nilsberth et al., 2001; Herzig et al., 2004).

The inflammation component although not dominating was represented in the TNF-signaling pathway, TNF being a potent pro-inflammatory cytokine. In particular, the leukocytes’ activation and recruitment genes (*CCL2*, *CXCL2*, and *CSF1*) are present and could indicate a vascular inflammation and endothelial cell activation, similarly to AD dysregulation (Grammas and Ovase, 2001). PI3k/Akt signaling, also part of the TNF super-family, is involved in AD affecting endothelial cell viability and angiogenesis (Grammas et al., 2011).

Lastly, the heat shock proteins (HSPs) family was highly represented in the top up-regulated genes, in particular HSP70 (HSPA family). HSPs are commonly up-regulated in AD (Koren et al., 2009) but also in PD, PSP, and FTLD-Tau (Milanesi and Pilotto, 2014). They are central to many mechanisms and likely involved in response to ER stress and protein misfolding but can also have an anti-apoptotic role (via JNK, MAPK, ERK, and PARP-1 signaling) and modulate ECM (via focal adhesion and Akt signaling). Small HSPs (sHSP, HSPB family) contribute to neuropathology by actively triggering inflammatory reactions in AD and in HCHWA-D (Wilhelmus et al., 2006b, 2009). Noticeably, the top up-regulated sHSPs in our study HSP27 (*HSPB1*) and αB-crystallin (*CRYAB*), were not associated with CAA in HCHWA-D but were already suggested to be efficient chaperone maintaining the peptide in an oligomeric state not trapped into aggregates (Wilhelmus et al., 2006a, 2009).

### Overlap With APP-E693Q Mouse Model Pathways

We compared transcriptome changes in HCHWA-D human brain tissue and the APP-E693Q mouse model in order to isolate mutation-associated primary dysregulation with the mouse model representing an early stage of the disease before CAA appearance, while the human post-mortem samples are the end stage of the disease. The identified ECM-related pathway overlap is striking.

Genes involved in cellular aerobic respiration were not affected in the transcriptome of 12-month APPE-693Q mice but phagosomal system genes were predominantly up-regulated, possibly indicating an early compensatory mechanism to remove the excess of toxic proteins. Consistently, an age-dependent lysosomal dysfunction was recently described in the APP-E693Q mice, with a higher lysosomal count in the entorhinal cortex of the 12-month mice and autophagosomal/autolysosomal protein level increases only in 24-month old mice, leading to an inflammatory reaction and neuronal loss (Kaur et al., 2017). In HCHWA-D, the lysosomal pathway was significantly down-regulated probably representing an end-stage state of the disease.

Although we did not find a significant overlap in the complement and coagulation cascades pathway, *SERPINE1* and *SERPING1* were commonly up-regulated in HCHWA-D and APP-E693Q indicating an early altered balance between thrombosis and fibrinolysis. *SERPING1* (C1-inhibitor) is controlling complement activation, blood coagulation, fibrinolysis, and generation of kinins. C1-inhibitors are neuroprotective after ischemic stroke (Heydenreich et al., 2012) and traumatic brain injury (Albert-Weissenberger et al., 2014) via important anti-inflammatory and antithrombotic mechanisms. On the other hand, *SERPINE1* plays a major role in pro-thrombotic conditions, regulating tissue-type plasminogen activator (tPA) and urokinase-type plasminogen activator (uPA) activity. *SERPINE1* gene expression was found up-regulated in AD brain (Magistri et al., 2015) as well and could be a plasmatic biomarker for the early detection and diagnosis of AD (Oh et al., 2014).

## Conclusion

An increase in ECM-related pathways was identified in HCHWA-D and could be, based on the APP-E693Q mice, involved in an early dysregulation inducing pro-fibrotic mechanisms. The mitochondrial dysfunction in HCHWA-D might be a consequence of impaired lysosomal/phagosomal function, in link with the proteinopathies. Alternatively, the formation of toxic oligomeric Aβ species described in the APP-E693Q mice could trigger oxidative stress affecting the mitochondrial compartment. The formation of toxic oligomeric Aβ species in HCHWA-D and their role of oxidative stress on neuronal, vascular, and perivascular cell would require further investigations.

## Data Availability

Sequence data and count table generated during the current study have been deposited at the European genome-phenome Archive (EGA, http://www.ebi.ac.uk/ega/) which is hosted at the European Bioinformatics Institute (EBI) under EGA accession number EGAD00001003806. Data are available upon approval of the Data Access Committees (EGAC00001000771).

## Author Contributions

LM, WvR-M, LvdW, SK, HM, HB, and JL designed the experiments. LM, LvdG, and EdM performed the experiments. HB supervised the sequencing. HM and SK processed, controlled, and generated the datasets. LM interpreted the datasets and wrote the manuscript. WvR-M and LvdW supervised and co-wrote the manuscript. KH, SvD, MvB, P‘tH, and SvdM critically revised the manuscript. All authors contributed to manuscript revision and read and approved the submitted version.

## Conflict of Interest Statement

The authors declare that the research was conducted in the absence of any commercial or financial relationships that could be construed as a potential conflict of interest.

## Footnotes

1 http://biopet-docs.readthedocs.io/en/latest/releasenotes/release_notes_0.6.0/
