# Supplementary material for: Brain Transcriptomic Analysis of Hereditary Cerebral Hemorrhage With Amyloidosis-Dutch Type
Source: Front Aging Neurosci. 2018 Apr 13;10:102. doi: 10.3389/fnagi.2018.00102 (PMC5908973; doi:10.3389/fnagi.2018.00102)
Supplement: Supplementary file 1 [file Data_Sheet_1.DOCX]

Supplementary Method

Brain transcriptomic analysis of hereditary cerebral hemorrhage with amyloidosis–Dutch type

Laure Grand Moursel^*^, Willeke M.C. van Roon-Mom, Szymon M. Kiełbasa, Hailiang Mei, Henk P.J. Buermans, Linda M. van der Graaf, Kristina M. Hettne, Emile J. de Meijer, Sjoerd G. van Duinen^3^, Jeroen F.J. Laros, Mark A. van Buchem, Peter A.C. ‘t Hoen, Silvère M. van der Maarel, Louise van der Weerd.

*** Correspondence:** [L.Grand_Moursel@lumc.nl](mailto:L.Grand_Moursel@lumc.nl)

# Brain tissue processing, RNA isolation, library preparation and sequencing

Frozen brain tissue was cut with a sliding microtome (Leica SM2010R) and sections were collected in ceramic MagNA-lyser beads tubes (Roche) for both RNA and protein extraction. Tissue homogenization was performed using a Bullet Blender (Next Advance). RNA samples were extracted with the Aurum Total RNA Mini Kit (Biorad), including an on-column DNaseI treatment. cDNA depleted RNA (500ng total RNA input) was fragmented to 150-200 nucleotides in first strand buffer for 3 minutes at 94°C. Random hexamer primed first strand was generated in presence of dATP, dGTP, dCTP and cTTP. Second strand was generated using dUTP instead of dTTP to tag the second strand. Subsequent steps to generate the sequencing libraries were performed with the KAPA HTP Library Preparation Kit for Illumina sequencing with minor modifications, i.e., after indexed adapter ligation to the dsDNA fragments, the library was treated with USER enzyme (M5505L, NEB) in order to digest the second strand derived fragments.

# RNA-Seq quality controls

We performed a sample concordance check based on the detected Single nuclear polymorphisms (SNPs) in all 36 RNA-Seq samples to ensure all these 36 samples are paired properly to 18 human subjects. First, we used VarScan (RRID:SCR_006849, v2.3.7) with default settings to call SNPs on the BAM files generated by GSNAP. Then, only sites (1394 in total) where all 36 RNA-Seq samples had sufficient coverage for genotyping were selected. Genotype concordance between frontal and occipital samples was checked using these variants. Gender concordance was verified with RPKM values of four genes located on chromosome Y [Protein Kinase, Y-Linked (PRKY), Ubiquitin Specific Peptidase 9, Y-Linked (USP9Y), Ubiquitously Transcribed Tetratricopeptide Repeat Containing, Y-Linked (UTY) and Zinc Finger Protein, Y-Linked (ZFY)]. Presence of the HCHWA-D mutation (NG_007376.1:g.283965G>C) was confirmed in HCHWA-D samples by manual inspection of the original BAM file using Integrative Genomics Viewer (RRID:SCR_011793, v2.3.80). Median 5’-3’ bias was calculated with Picard RnaSeqMetrics^[[1]](#footnote-1)^ which reports the ratio of coverage at the 5 prime end to the 3 prime end, based on the 1000 most highly expressed transcripts.

# Quantitative RT-PCR

cDNA was synthesized directly after RNA extraction with the Transcriptor First Strand cDNA Synthesis Kit (Roche) using Random Hexamer primers at 65 °C. Primers were designed with Primer3 Plus software (Untergasser et al., 2012). The qPCR was performed in a 384 well plate, pre-spotted with primer pairs, using 6 ng of cDNA per well in a PCR master mix containing the EvaGreen qPCR dye (Biotum). All samples were run in duplicate along with three reference genes: Hydroxymethylbilane Synthase (*HMBS*), RibosomalProtein L22 (*RPL22*) and TATA-Box Binding Protein (*TBP*). The amplification was performed on a LightCycler 480 (Roche). Relative expression of the transcript levels was calculated using LinRegPCR v11.1 (Ruijter et al., 2009), normalized per sample with two of the reference genes (*HMBS* and *RLP22*) and transcript levels were calculated per patient (combined frontal and occipital data) with the Geomean of the repeats (8 points). The third reference gene (*TBP*) was used to check the normalization efficiency and the inter-plate variance.

# Western blot

Tissue homogenization from frozen brain sections was performed using Bullet Blender (Next Advance). Proteins were extracted in lysis buffer (Tris 50mM pH7.5, Tritonx100 1%) containing 1x proteinase inhibitor (Complete mini, Roche). Supernatant from centrifugation at 14000 rpm, 4°C was collected and total protein concentration was measured using the BCA method. Samples were adjusted to a final concentration of 2µg/µL in Laemmli buffer, denatured 10min at 95°C and stored at -20°C. Protein samples (28 µg per lane) were run on a 4–15% Mini-PROTEAN™ TGX Stain-Free™ Protein Gels (Bio-Rad), transferred to nitrocellulose membrane with Trans-Blot system (Bio-Rad), blocked for 1 hour in blocking buffer (5% milk in 0.1 % Tween 20 in Tris Buffer Saline pH 7.4); probed with primary antibodies overnight at 4°C in blocking buffer with anti-HSP70 (1:1500, Santa Cruz Biotechnology Cat# sc-24, RRID:AB_627760) and anti-β-actin (1:5000, Abcam Cat# ab6276, RRID:AB_2223210). Conjugated secondary antibodies anti-mouse IRDye®680RD (LI-COR Biosciences Cat# 926-68072, RRID:AB_10953628) and anti-rabbit IRDye®800CW (LI-COR Biosciences Cat# 926-32213, RRID:AB_621848); diluted at 1:5000 in blocking buffer were added for 1.5h at room temperature. The signal was measured with the Odyssey CLx (RRID:SCR_014579). Quantification of the bands was done with Image Studio Lite (Image Studio Lite, v5.2). HSP70 signal was normalized for the β-actin signal.

# Additional references

Ruijter, J. M., Ramakers, C., Hoogaars, W. M. H., Karlen, Y., Bakker, O., van den Hoff, M. J. B., et al. (2009). Amplification efficiency: Linking baseline and bias in the analysis of quantitative PCR data. *Nucleic Acids Res.* 37, e45. doi:10.1093/nar/gkp045.

Untergasser, A., Cutcutache, I., Koressaar, T., Ye, J., Faircloth, B. C., Remm, M., et al. (2012). Primer3--new capabilities and interfaces. *Nucleic Acids Res.* 40, e115. doi:10.1093/nar/gks596.

1. http://broadinstitute.github.io/picard/ [↑](#footnote-ref-1)
