# Supplementary material for: Brain Transcriptomic Analysis of Hereditary Cerebral Hemorrhage With Amyloidosis-Dutch Type
Source: Front Aging Neurosci. 2018 Apr 13;10:102. doi: 10.3389/fnagi.2018.00102 (PMC5908973; doi:10.3389/fnagi.2018.00102)
Supplement: Supplementary file 2 [file Data_Sheet_2.DOCX]

Supplementary Tables

Brain transcriptomic analysis of hereditary cerebral hemorrhage with amyloidosis–Dutch type

Laure Grand Moursel^*^, Willeke M.C. van Roon-Mom, Szymon M. Kiełbasa, Hailiang Mei, Henk P.J. Buermans, Linda M. van der Graaf, Kristina M. Hettne, Emile J. de Meijer, Sjoerd G. van Duinen^3^, Jeroen F.J. Laros, Mark A. van Buchem, Peter A.C. ‘t Hoen, Silvère M. van der Maarel, Louise van der Weerd.

*** Correspondence:** [L.Grand_Moursel@lumc.nl](mailto:L.Grand_Moursel@lumc.nl)

**Supplementary Table 1:** Primer list used for qPCR

| Genebank Acc. Num. | Name | Primers | Target |
| --- | --- | --- | --- |
| NM_005345 | HSPA1A | 5’-ATAAAAGCCCAGGGGCAAG-3’  5’-GGGAGTCACTCTCGAAAAAGG-3’ | Exonic (intronless) |
| NM_002523 | NPTX2 | 5’-TGATCCTTGGACAAGAGCAG-3’  5’-ATTTCTTGTGCGCGAAGG-3’ | Intron spanning (exons 4_5) |
| NM_024411 | PDYN | 5’-TGCTGTAAAGACCCAGGATG-3’  5’-AGACAGAAAGCTCTGGCATCTC-3’ | Intron spanning (exons 2_3) |
| NM_005276 | GPD1 | 5’-ACACGCAGCATGAGAATGTC-3’  5’-CACAGATCTTGCCGATGAAC-3’ | Intron spanning (exons 2_3) |
| NM_001171171 | CX3CR1 | 5’-TGGTAAAGTCTGAGCAGGACAG-3’  5’-TAACAGGCCTCAGCCAAATC-3’ | Intron spanning (exons 1_2) |
| NM_003194 | TBP | 5'-CGCCGAATATAATCCCAAGC-3'  5'-GAAAATCAGTGCCGTGGTTC-3' | Intron spanning (reference gene) |
| NM_000983 | RPL22 | 5'-TCGCTCACCTCCCTTTCTAA-3'  5'-TCACGGTGATCTTGCTCTTG-3' | Intron spanning (reference gene) |
| NM_000190 | HMBS | 5'-GCAACGGCGGAAGAAAA-3'  5'-CGAGGCTTTCAATGTTGCC-3' | Intron spanning (reference gene) |

**Supplementary Table 2**: Details of HCHWA-D and control samples

| Diagnosis | Samples^a^  (frontal/occipital) | RIN | Median 5’ to 3’ bias |
| --- | --- | --- | --- |
| NDC | S_13 / S_14 | 7.7 / 6.3 | 0.7 / 0.5 |
| NDC | S_17 / S_18 | 8.7 / 7.9 | 7.9 / 8.4 |
| NDC | S_3 / S_4 | 8.2 / 7.5 | 0,7 / 0,6 |
| NDC | S_1 / S_2 | 8.3 / 7.2 | 1,3 / 1,1 |
| NDC | S_11 / S_12 | 8.1 / 7.3 | 1.0 / 0.9 |
| NDC | S_5 / S_6 | 8.6 / 7.4 | 0.6 / 0.6 |
| NDC | S_7 / S_8 | 8.8 / 6.5 | 5.7 / 0.7 |
| NDC | S_9 / S_10 | 8.9 / 8.8 | 0.8 / 0.7 |
| NDC | S_15 / S_16 | 8.5 / 6.3 | 0.6 / 0.6 |
| HCHWA-D | S_19 / S_20 | 6.2 / 6.6 | 13.9 / 4.8 |
| HCHWA-D | S_21 / S_22 | 6.3 / 6.9 | 7.9 / 5.2 |
| HCHWA-D | S_31 / S_32 | 6.3 / 6.4 | 3,9 / 5,8 |
| HCHWA-D | S_25 / S_26 | n.a. | 0,6 / 0,9 |
| HCHWA-D | S_27 / S_28 | 6.2 / 5.2 | 0,6 / 8,1 |
| HCHWA-D | S_33 / S_34 | 8.5 / 8.1 | 0,9 / 0,6 |
| HCHWA-D | S_35 / S_36 | 6.3 / 6.6 | 3,9 / 5,7 |
| HCHWA-D | S_29 / S_30 | 5.7 / 5.7 | 0,7 / 0,7 |
| HCHWA-D | S_23 / S_24 | 7.8 / 5.1 | 10,1 / 6,8 |

NDC non-demented control, HCHWA-D hereditary cerebral hemorrhage with amyloidosis-Dutch type, n.a. not available

^a^ Samples code used for RNA-Seq
