# Supplementary material for: Brain Transcriptomic Analysis of Hereditary Cerebral Hemorrhage With Amyloidosis-Dutch Type
Source: Front Aging Neurosci. 2018 Apr 13;10:102. doi: 10.3389/fnagi.2018.00102 (PMC5908973; doi:10.3389/fnagi.2018.00102)
Supplement: Supplementary file 3 [file Data_Sheet_3.docx]

Supplementary Figures

Brain transcriptomic analysis of hereditary cerebral hemorrhage with amyloidosis–Dutch type

Laure Grand Moursel^*^, Willeke M.C. van Roon-Mom, Szymon M. Kiełbasa, Hailiang Mei, Henk P.J. Buermans, Linda M. van der Graaf, Kristina M. Hettne, Emile J. de Meijer, Sjoerd G. van Duinen, Jeroen F.J. Laros, Mark A. van Buchem, Peter A.C. ‘t Hoen, Silvère M. van der Maarel, Louise van der Weerd.

*** Correspondence:** [L.Grand_Moursel@lumc.nl](mailto:L.Grand_Moursel@lumc.nl)


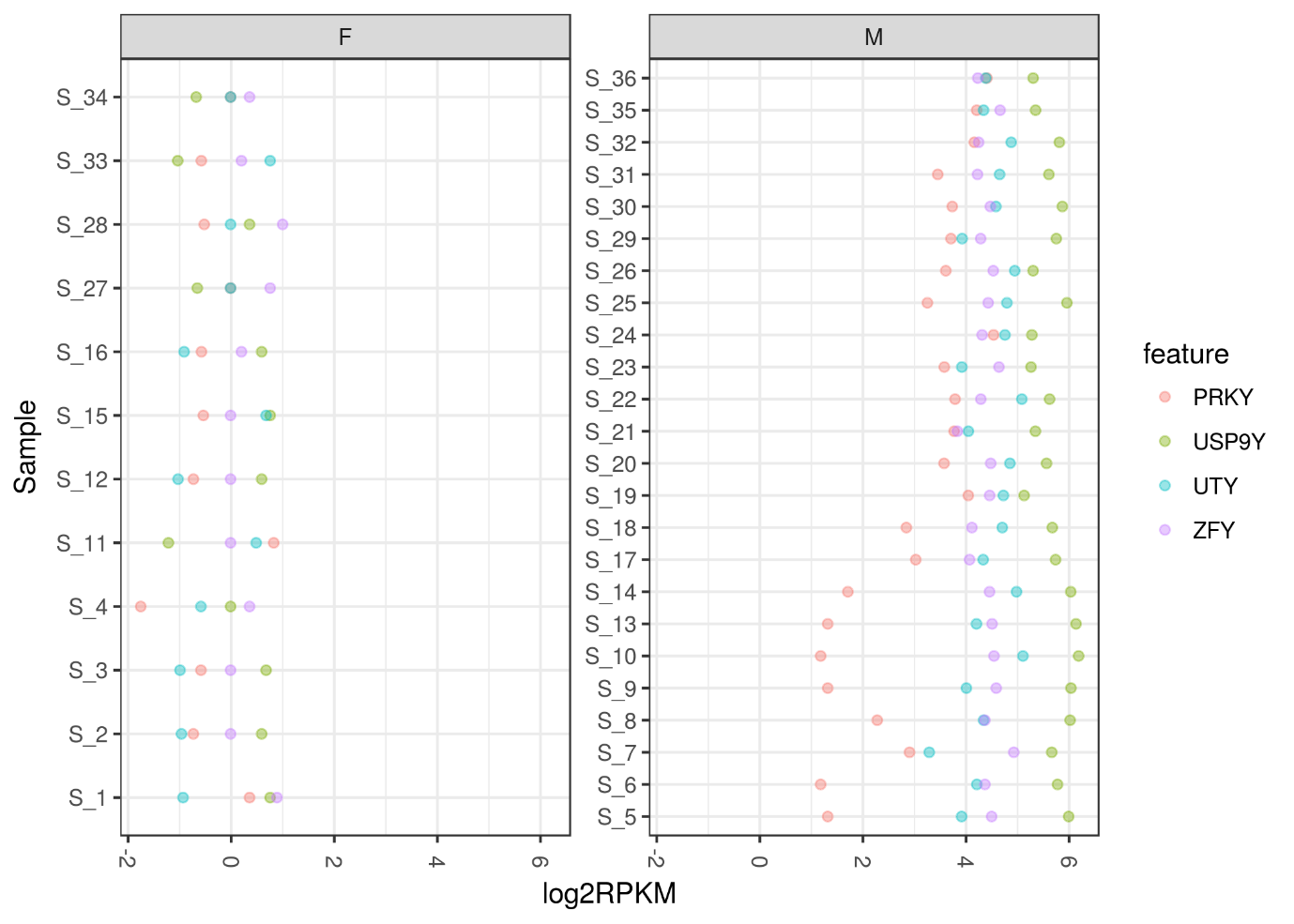


**Supplementary Figure 1:** Gender concordance verified with log2RPKM expression values of four genes located on chromosome Y [Protein Kinase, Y-Linked (PRKY), Ubiquitin Specific Peptidase 9, Y-Linked (USP9Y), Ubiquitously Transcribed Tetratricopeptide Repeat Containing, Y-Linked (UTY) and Zinc Finger Protein, Y-Linked (ZFY)].


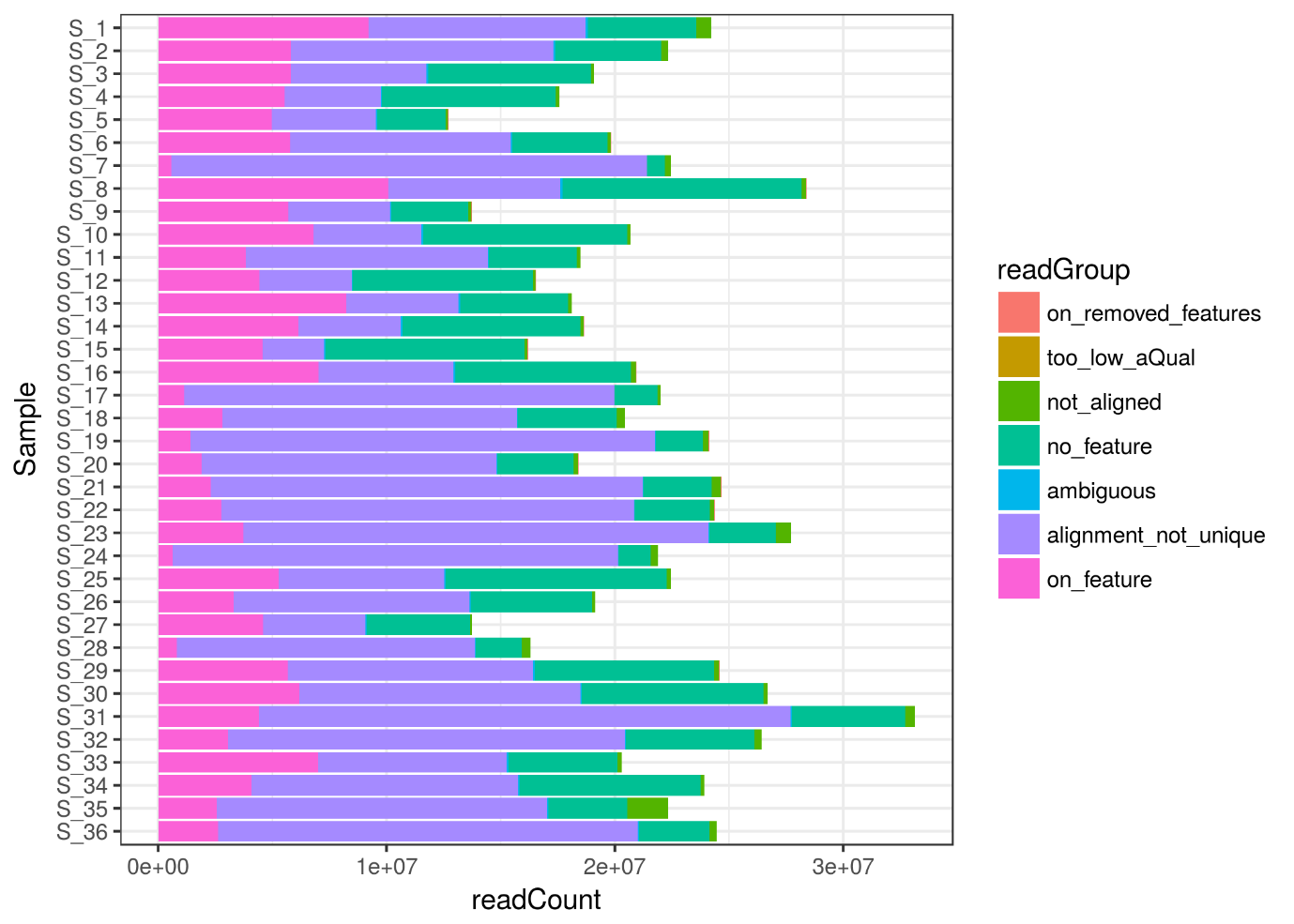


**Supplementary Figure 2:** Alignment summary. ‘’on feature’’ reads were reported into the count table; ‘’alignment not unique’’ reads were discarded (higher proportion in HCHWA-D samples), ‘’no feature’’ is non-coding RNA.

**Supplementary Figure 3:** Positive correlation between the median 5'-3' bias and high GC content gene fragments (*p*<0.0001 as determined by a two-tailed Pearson statistic test). The median 5'-3' bias per sample is plotted against the sum of the relative number of fragments with gene GC content between 75% and 100%.


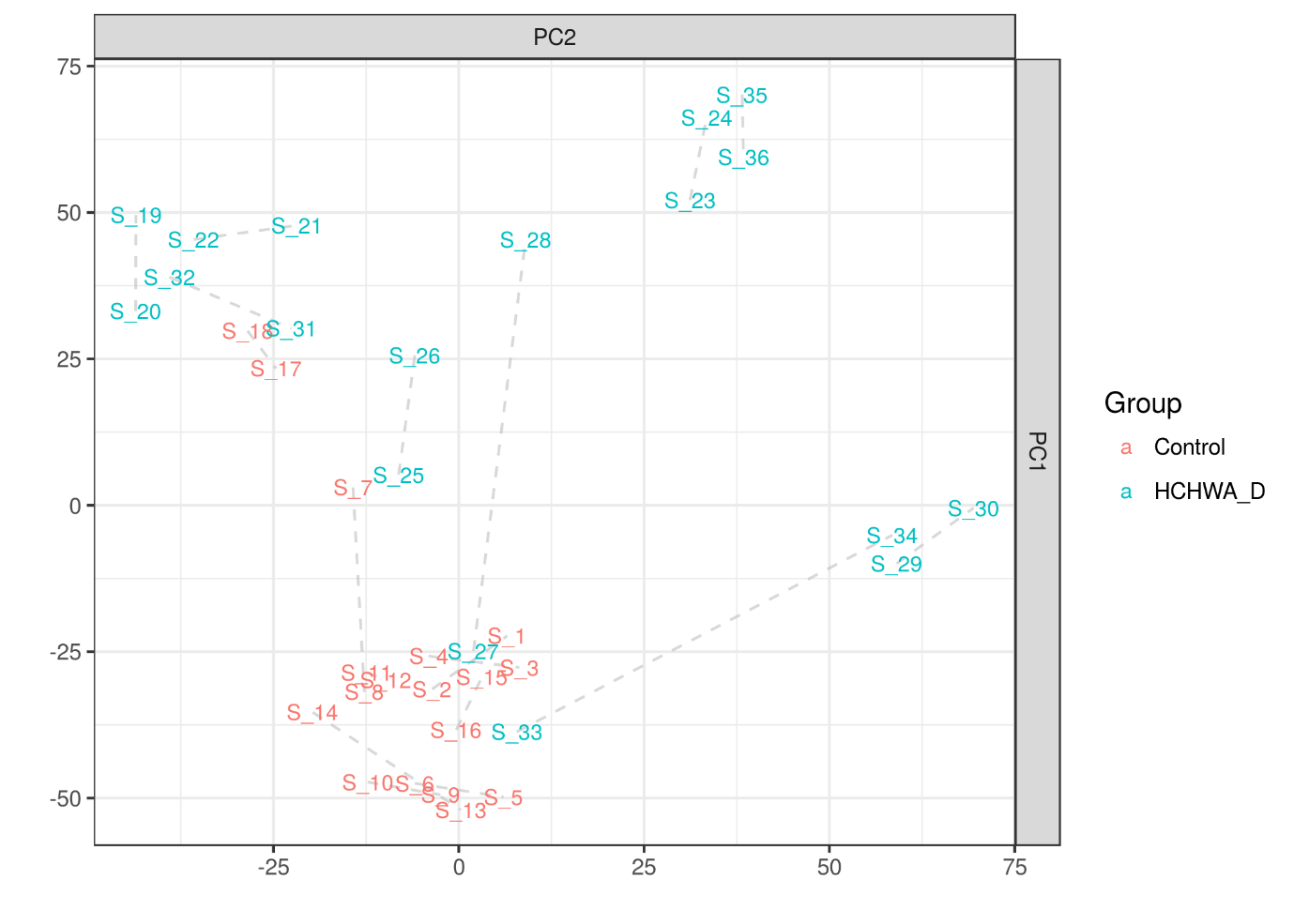


**Supplementary Figure 4:** Principal Component Analysis (PCA) per groups. Frontal and occipital cortex from same individual are linked by dashed lines.


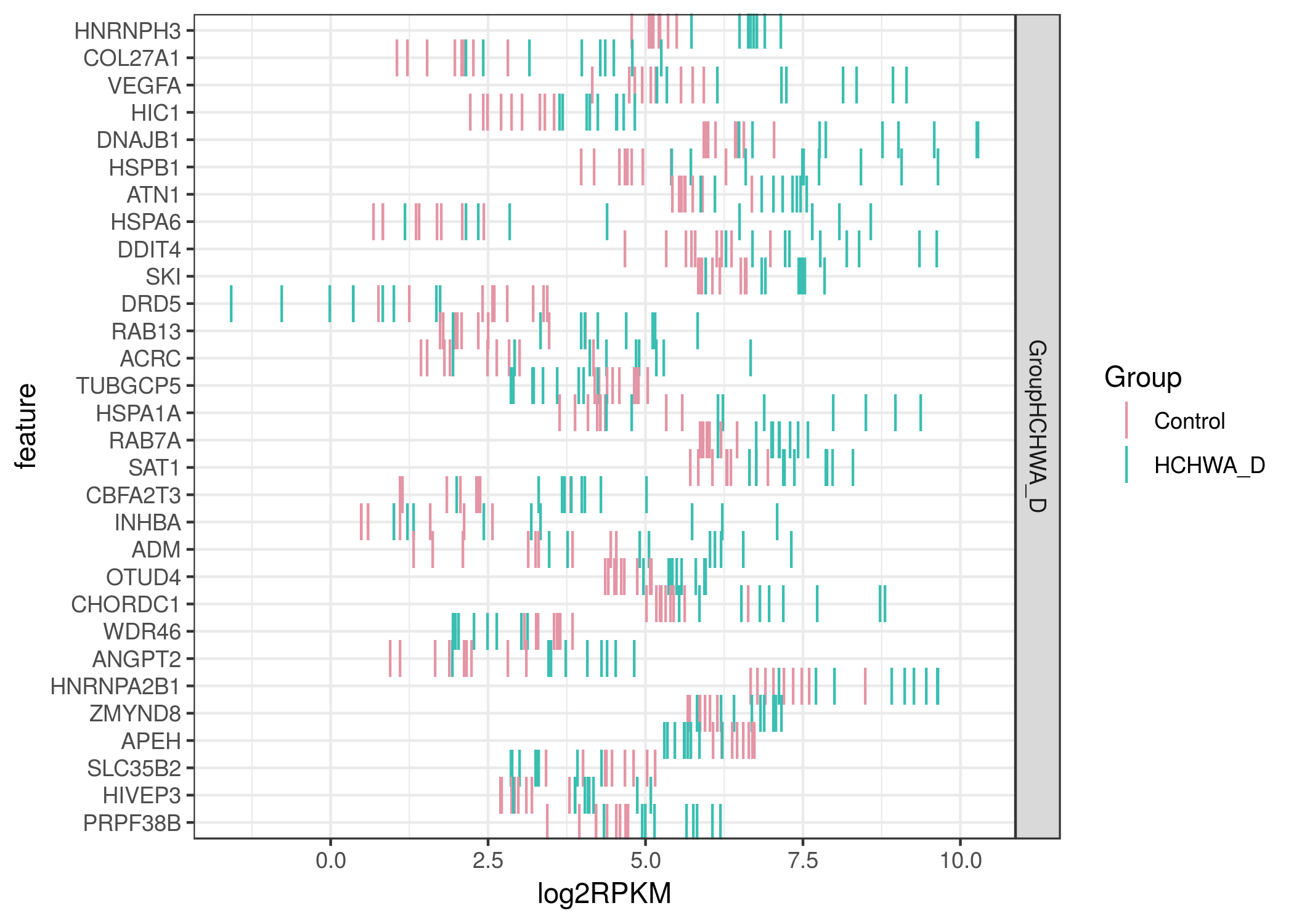


**Supplementary Figure 5:** Top HCHWA-D DEG ranked on significance (FDR) with log2RPKM expression values.


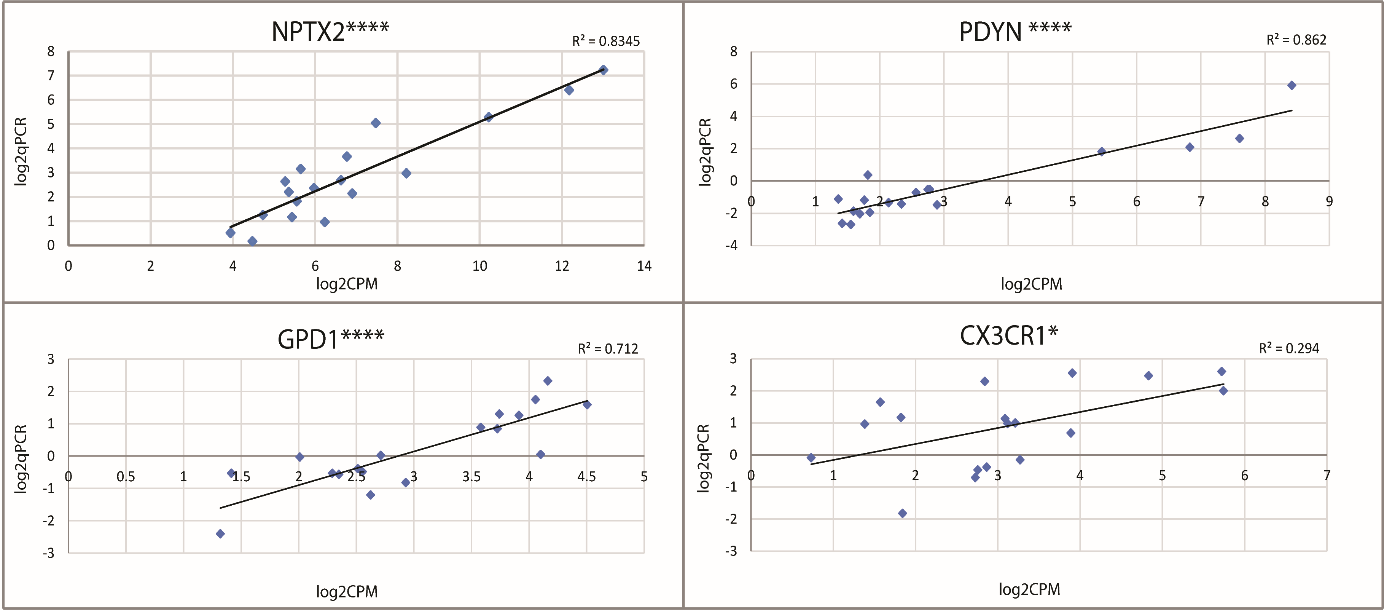


**Supplementary Figure 6** Correlation of expression levels between the RNAseq and the qPCR data (mean per patient in log2 scale) for NPTX2, PDYN, GPD1 and CX3CR1 (NPTX2 and PDYN were up-regulated; GPD1 and CX3CR1 were down-regulated). **p*<0.05, ***p*<0.01, ****p*<0.001 and *****p*<0.0001 as determined by a two-tailed Pearson statistic test.


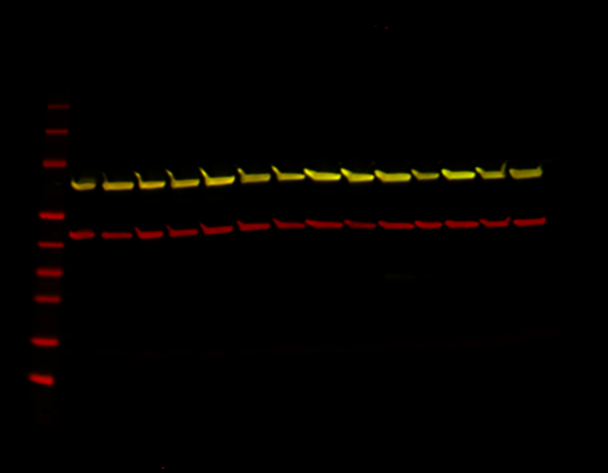


100

55

40

25

35

180

130

10

15

kDa

Control

HCHWA-D

HSP70

β-actin

**Supplementary Figure 7:** Western Blot picture in double channel colors including the β-actin loading control on the same blot (non-cropped picture; 680nm in red and 800nm in green).


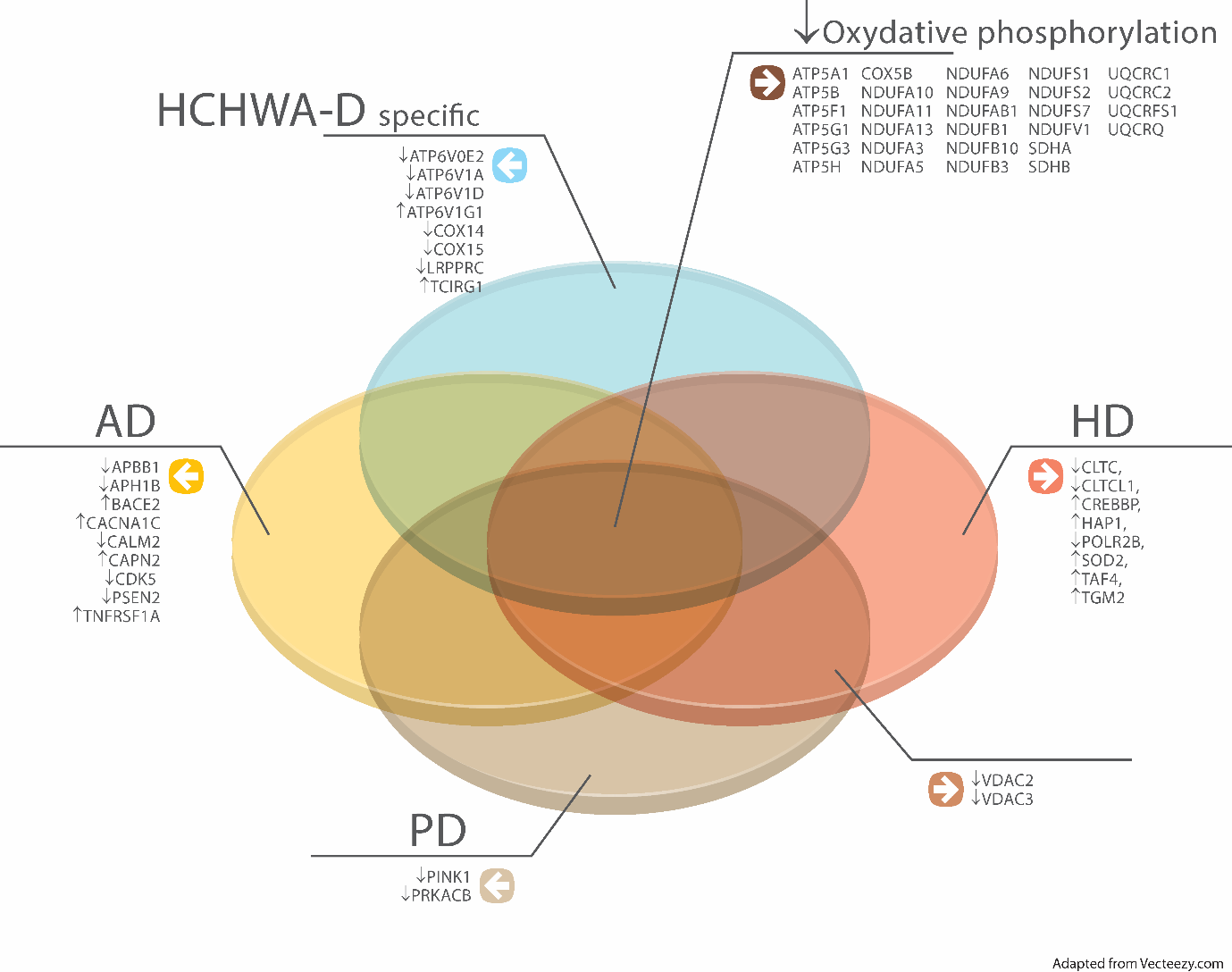


**Supplementary Figure 8:** Venn diagram representing the overlap in oxidative phosphorylation pathway (in KEGG database), the genes found in PD, AD, and HD neurodegenerative diseases and the genes specifically dysregulated in HCHWA-D. Arrows in front of gene name indicates whether the genes were up- or down-regulated in HCHWA-D transcriptome.


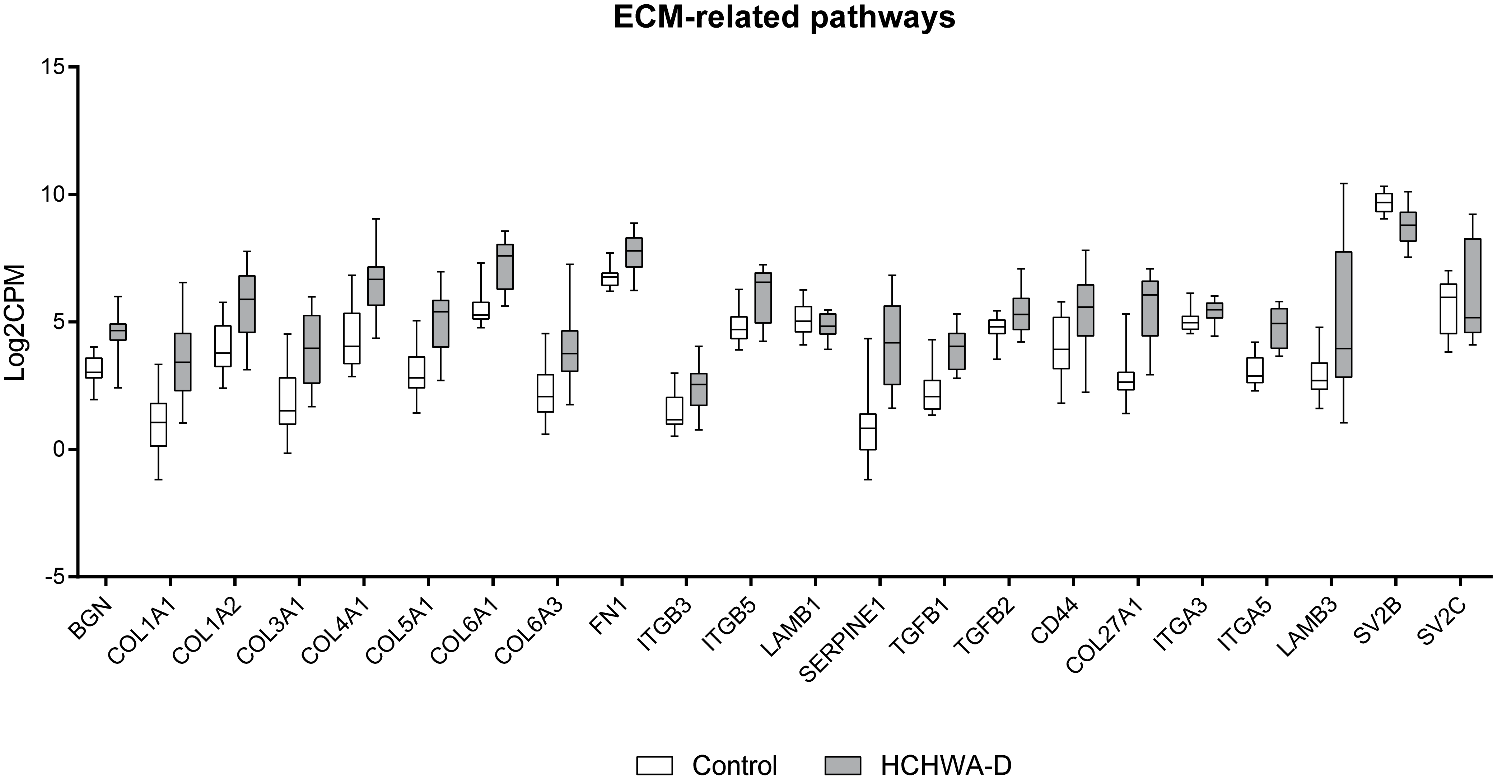


**Supplementary Figure 9:** Expression values (log2CPM) in boxplots of identified genes from ECM-related pathway genes (GSEA with gene list ranked on FC; DEG subset FDR<0.05).


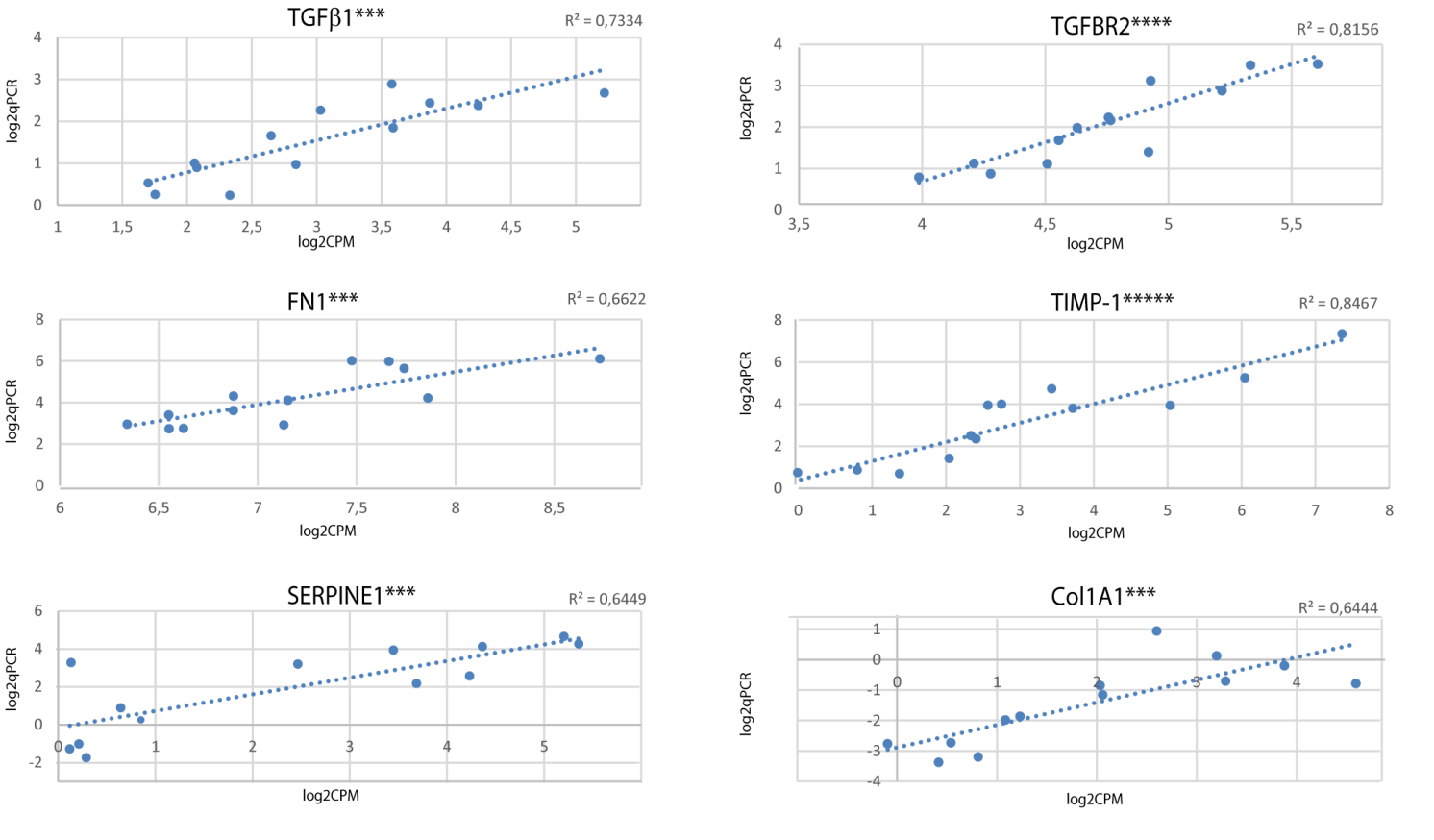


**Supplementary Figure 10:** Correlation of expression levels between the RNAseq (log2CPM; mean per patient) and the qPCR data (mean per patient in log2 scale) on a subset of patients (HCHWA-D n=6, Controls n=7) for up-regulated genes associated with ECM-related pathways. qPCR data with the exception of TIMP-1**^#^** were previously published (Grand Moursel et al., 2017). **p*<0.05, ***p*<0.01, ****p*<0.001, *****p*<0.0001 and ******p*<0.00001 as determined by a two-tailed Pearson statistic test.

| Genebank Acc. Num. | Name | ^#^Primers | Target |
| --- | --- | --- | --- |
| NM_003254 | TIMP-1 | 5'-AATTCCGACCTCGTCATCAG-3'  5'-CATCCCCTAAGGCTTGGAAC-3' | Intron-spanning (exons 2-3-4) |

# Reference

Grand Moursel, L., Munting, L. P., van der Graaf, L. M., van Duinen, S. G., Goumans, M.-J. T. H., Ueberham, U., et al. (2017). TGFbeta pathway deregulation and abnormal phospho-SMAD2/3 staining in hereditary cerebral hemorrhage with amyloidosis-Dutch type. *Brain Pathol.*, 1–12. doi:10.1111/bpa.12533.
